# A token-pruning framework enables efficient representation of the human genome for RNA modification analysis

**DOI:** 10.1093/bioinformatics/btag621

**Published:** 2026-09-01

**Authors:** Wenjia Gao, Junlei Yu, Junru Jin, Jiajie Cai, Ke Qiu, Shun Zhang, Jianbo Qiao, Leyi Wei

**Affiliations:** School of Software, Shandong University, Jinan, China; School of Software, Shandong University, Jinan, China; School of Software, Shandong University, Jinan, China; School of Software, Shandong University, Jinan, China; School of Software, Shandong University, Jinan, China; School of Software, Shandong University, Jinan, China; School of Software, Shandong University, Jinan, China; Engineering Research Centre of Applied Technology on Machine Translation and Artificial Intelligence, Macao Polytechnic University, Macao SAR, China; Joint SDU-NTU Centre for Artificial Intelligence Research (C-FAIR), Shandong University, Jinan, China

## Abstract

**Motivation:**

Modelling long genomic sequences remains challenging due to extreme sequence length, high redundancy, and the need for biological interpretability. Although Transformer-based architectures have achieved strong performance across genomic tasks, their high computational cost and reliance on fixed tokenization strategies limit their scalability and ability to focus on biologically informative regions.

**Results:**

We propose ATSFormer, a token-pruning Transformer framework for efficient and biologically informed genomic sequence modelling. ATSFormer incorporates an attention-guided and parameter-free Adaptive Token Sampling (ATS) module into Transformer layers. Guided by attention-derived importance scores, ATS dynamically retains informative tokens while probabilistically discarding redundant ones, thereby reducing sequence length, FLOPs, and memory usage without introducing additional learnable parameters or extra training procedures. Importantly, the retained tokens correspond to key contributors to model predictions, enabling ATSFormer to highlight biologically meaningful sites and sequence motifs. We evaluated ATSFormer on four benchmark RNA modification datasets derived from RMVar 2.0, covering A-to-I, m1A, m5C, and m7G. Experimental results show that ATSFormer consistently outperforms existing state-of-the-art methods while achieving substantial computational savings. Furthermore, structural analysis using AlphaFold3 supports the biological relevance of the motifs identified by ATSFormer.

**Availability and implementation:**

The source data and code are freely available at GitHub (https://github.com/1gao2/ATSFormer) and Zenodo (https://doi.org/10.5281/zenodo.21813541).

## 1 Introduction

The human genome, spanning roughly three billion base pairs, encodes instructions that guide growth, development, and survival in every cell (Lander *et al*. 2001, Avsec *et al*. 2021). This vast repository of genetic information is essential for understanding molecular functions and disease mechanisms. Despite decades of research, much of the genome remains poorly understood. Only a small fraction of genomic regions are functionally annotated for protein coding or gene regulation, while the majority remains largely uncharted (Chen *et al*. 2022).

In recent years, computational methods have become essential for interpreting the human genome (Dalla-Torre *et al*. 2025). From predicting gene function to identifying regulatory elements, these models provide key insights into how genetic sequences shape biological outcomes. Deep learning (DL) approaches, especially models such as ERNIE-RNA (Yin *et al*. 2025), GROVER (Sanabria *et al*. 2024), and other NLP-inspired architectures (Zhou *et al*. 2019), have shown remarkable ability to capture complex patterns in genomic data, often surpassing traditional methods in tasks such as sequence modification prediction, variant effect prediction, and gene expression modelling. NLP-inspired techniques are particularly effective (Zhou 2022): just as language models parse word sequences to extract meaning, DL models can parse nucleotide sequences to discover biologically relevant features.

A critical step in applying NLP-inspired models to genomic sequences is tokenization. Character N-gram methods (Nguyen *et al*. 2023) split sequences into fixed-length fragments to capture local patterns, while Byte Pair Encoding (BPE) (Sennrich *et al*. 2016) generates variable-length tokens by merging frequent character pairs, thereby increasing representation flexibility and information density. Although these methods have been effective in language tasks, they are less suitable for genomics because genomic motifs are often degenerate, subtly variable across sites, and highly context-dependent, making them difficult to capture with conventional tokenization schemes (Wu *et al*. 2025).

Since 2019, Transformer models have gained wide adoption in genomics (Karollus *et al*. 2024). Models such as Nucleotide Transformer (Dalla-Torre *et al*. 2025) are pretrained on large-scale genomic data to learn context-aware nucleotide representations for downstream tasks such as variant effect prediction. ERNIE-RNA (Yin *et al*. 2025) further extends BERT-style RNA modelling by incorporating base-pairing-informed attention bias into the self-attention mechanism, thereby enhancing the representation of RNA structural and functional features. GROVER (Sanabria *et al*. 2024) is another DNA language model trained with masked token prediction, using a specialized genomic vocabulary and demonstrating strong capability in learning sequence context. However, these models remain limited in handling long sequences, while also incurring high training costs and offering limited interpretability (Jin *et al*. 2022).

RNA modification prediction provides a biologically meaningful benchmark for evaluating whether advanced tokenization strategies can model long genomic sequences while capturing both local and long-range context. Among RNA modifications, adenosine-to-inosine (A-to-I) RNA editing is particularly important and is catalysed by ADAR enzymes (Nishikura 2010, Eisenberg and Levanon 2018, Min *et al*. 2026). A-to-I editing recodes RNA by converting adenosine into inosine, which is interpreted as guanosine during translation, thereby expanding transcriptomic and proteomic diversity. This process is critical in neurodevelopment, immune regulation, and tumourigenesis, with dysregulation implicated in numerous diseases (Nishikura 2016, Jain *et al*. 2019, Marônek *et al*. 2025). Accurate identification of RNA modification sites requires models to capture both local motif features and long-range contextual signals, challenging conventional fixed-length or coarse-grained tokenization schemes (Chen *et al*. 2024).

Existing RNA modification prediction methods have explored diverse modelling strategies. Traditional machine learning methods, such as iMRM (Liu and Chen 2020), use an XGBoost-based framework with k-mer, physicochemical, and structural features to predict multiple RNA modifications. RDDSVM (Tac *et al*. 2021) applies support vector machines to identify A-to-I editing sites based on triplet composition of flanking sequences. Deep learning-based methods have also been developed for this task. PreAIS (Fang *et al*. 2025) combines DNN and CNN architectures with k-mer features for accurate A-to-I site prediction. MRM-BERT (Wang and Zhou 2024) fine-tunes a pretrained DNABERT model and integrates convolutional features to predict multiple RNA modifications. MultiRM (Song *et al*. 2021) further employs an attention-based multi-label neural network to predict twelve common RNA modifications while identifying key sequence motifs. These methods collectively highlight different strategies for modelling RNA modifications, ranging from traditional machine learning to deep learning and pretrained Transformer architectures. However, most of them treat all tokens equally or rely on fixed-length representations, leading to high computational costs, limited biological interpretability, and reduced ability to focus on informative regions in long genomic sequences.

In addition to task-specific limitations, existing models still face challenges due to the large scale of genomes (de Boer and Taipale 2024). Standard tokenization, which treats each nucleotide as a token, produces extremely long inputs. This makes training and inference computationally expensive and limits the ability of models to capture key motifs. Although Transformer-based models such as Nucleotide Transformer (Dalla-Torre *et al*. 2025) and GROVER (Sanabria *et al*. 2024) have achieved strong performance in genomics, their quadratic computational complexity with respect to input length restricts their application to long sequences. In contrast, convolution-based methods can process long sequences, but they may be less effective in capturing complex long-range dependencies, further emphasizing the need for efficient token pruning.

Adaptive token pruning has been widely studied as an effective strategy for reducing Transformer computation. Adaptive Token Sampling introduces a differentiable and parameter-free module that uses attention-derived importance scores to adaptively sample informative tokens in Vision Transformers (Fayyaz *et al*. 2022). MADTP employs multimodal alignment to guide token pruning and dynamically adjusts the compression ratio across layers and input samples (Cao *et al*. 2024). More recently, Frequency-Aware Token Reduction separates high- and low-frequency tokens, preserving informative high-frequency tokens while aggregating low-frequency information to mitigate rank collapse and over-smoothing (Lee *et al*. 2026).

To address these challenges, we propose ATSFormer, a token-pruning Transformer framework for efficient and biologically informed genomic tokenization. By integrating Adaptive Token Sampling (ATS), ATSFormer dynamically identifies and removes less informative tokens. Importance scores are assigned via self-attention weights associated with the classification token. Adaptive sampling, implemented through multinomial or inverse transform methods, allows selective retention of biologically critical tokens while discarding low-importance ones. This enables scaling to larger genomic contexts, focusing computational resources on relevant regions and reducing input length and cost. Importantly, ATS preserves biological interpretability without sacrificing performance.

The main contributions of this work are:

- ATSFormer integrates an attention-guided discrete token sampling mechanism into Transformer layers without introducing additional trainable parameters, enabling the efficient processing of long genomic sequences.
- A parameter-free adaptive token sampling mechanism identifies and preserves biologically informative tokens, significantly reducing computational cost while maintaining predictive accuracy.
- ATSFormer enhances interpretability, as retained tokens correspond to core contributors to model decisions, highlighting key genomic sites and sequence motifs.
- Four large-scale benchmark datasets for RNA modification prediction—A-to-I, m1A, m5C, and m7G—are constructed based on RMVar 2.0 for comprehensive biological evaluation.
- Extensive experiments demonstrate that ATSFormer achieves state-of-the-art performance and efficiency, with identified motifs’ biological relevance further supported by AlphaFold3 structural analysis.

## 2 Materials and methods

### 2.1 The framework of ATSFormer

To enable efficient and biologically informed modelling of long human genomic sequences, we developed ATSFormer, a token-pruning Transformer framework with Adaptive Token Sampling (ATS). By introducing the ATS mechanism, ATSFormer can dynamically identify and retain informative tokens while discarding less relevant ones, thereby reducing input length and computational cost without compromising performance.

**Figure 1:**
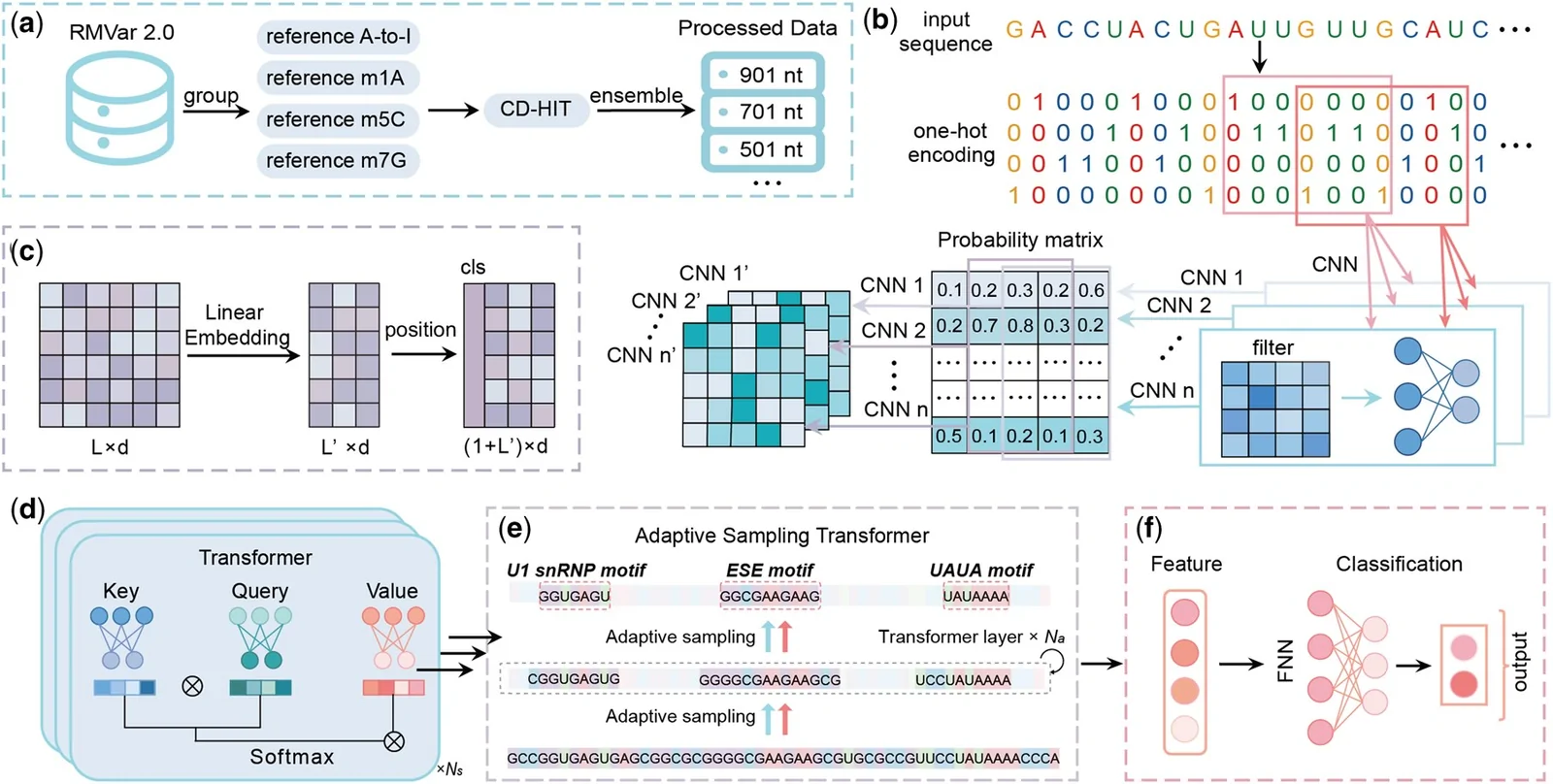
Overview of the ATSFormer framework. (a) Data construction pipeline. (b) Word-Detection Module. (c) Positional-Aware Token Embedding Module. (d) Standard Transformer Encoder Module. (e) Adaptive Token Sampling Transformer Module. (f) Classification Module.

As shown in Fig. 1, the ATSFormer framework comprises six main parts. We first constructed datasets for four RNA modification types and removed redundant sequences using CD-HIT (Fig. 1a). The resulting genomic sequences are then processed by a two-layer convolutional network to extract local features and perform adaptive downsampling, transforming the sequences into semantically meaningful ‘genomic words’ (Fig. 1b). The Positional-Aware Token Embedding module projects these features into a dense space, prepends a learnable [CLS] token, and adds sinusoidal positional encoding to incorporate positional information (Fig. 1c). The standard Transformer layers compute full self-attention across all tokens to capture fundamental dependencies (Fig. 1d), while the subsequent ATS Transformer layers adaptively prune tokens based on attention-derived importance scores using multinomial or inverse transform sampling (Fig. 1e). Finally, the classification module aggregates the distilled information from the [CLS] token to produce accurate predictions (Fig. 1f). Together, these components allow ATSFormer to focus computational resources on biologically relevant regions, preserve interpretability, and capture both local motifs and long-range genomic context.

### 2.2 Word-detection module

The raw input sequence *X* of length *L* is first converted into a one-hot matrix $O\in {\left\{0,1\right\}}^{L\times 5}$, representing nucleotides A, C, G, T, and N (ambiguous base). Transposed to $O^{T}\in R^{5\times L}$, it is input to a two-layer 1D convolutional neural network (CNN) acting as an adaptive ‘word detector’ to extract local sequence patterns and perform content-aware downsampling.

The first convolution uses a larger kernel to capture broad motifs and reduce sequence length:


(1) $$H^{\left(1\right)}=\text{ReLU}\left(\text{BatchNorm}\left({\text{Conv}1D}_{7,3}\left(O^{T}\right)\right)\right)\in R^{N_{c}\times L^{\prime }}$$

with $N_{c}=128$ channels and $L^{\prime }=\left\lfloor (L+6-7)/3+1\right\rfloor$.

A second convolution with a smaller kernel refines features:


(2) $$H^{\left(2\right)}=\text{ReLU}\left(\text{BatchNorm}\left({\text{Conv}1D}_{3,1}\left(H^{\left(1\right)}\right)\right)\right)\in R^{N_{c}\times L^{\prime }}$$

This yields a condensed feature map $H^{\left(2\right)}$, which is transposed to $H\in R^{L^{\prime }\times N_{c}}$, representing the sequence as $L^{\prime }$ feature vectors, each summarizing a local genomic ‘word’.

### 2.3 Positional-aware token embedding module

Following processing by the Word-Detection Module, the genomic sequence is encoded into a sequence of local feature vectors. However, genomic function depends not only on local patterns but also on precise sequence order and long-range positional relationships. To incorporate this, we designed the Positional-Aware Token Embedding Module, which transforms local features into dense embeddings enriched with positional context for the Transformer encoder.

Specifically, the module receives $H\in R^{L^{\prime }\times N_{c}}$ from the Word-Detection Module ($L^{\prime }$ is the compressed sequence length, $N_{c}=128$). A linear layer projects each feature into the Transformer’s hidden dimension $d_{\text{model}}$:


(3) $$Z_{0}=HW_{e},\;W_{e}\in R^{N_{c}\times d_{\text{model}}}$$

yielding $Z_{0}\in R^{L^{\prime }\times d_{\text{model}}}$. A learnable [CLS] token $t_{\text{cls}}\in R^{1\times d_{\text{model}}}$ is prepended:


(4) $$Z_{\text{pre}}=[t_{\text{cls}};Z_{0}]\in R^{(L^{\prime }+1)\times d_{\text{model}}}$$

Sinusoidal positional encoding is then added to incorporate token position information:


(5) $$Z=Z_{\text{pre}}+P[:,:L^{\prime }+1],\;P\in R^{1\times L_{\text{max}}\times d_{\text{model}}}$$

The final output $Z\in R^{B\times (L^{\prime }+1)\times d_{\text{model}}}$ contains both local sequence semantics and global positional context, providing structured input for the Adaptive Token Sampling Transformer to capture long-range dependencies.

### 2.4 Adaptive token sampling transformer

The encoder of ATSFormer consists of N=6 layers in two stages. The first $N_{s}=3$ layers are standard Transformer encoder layers, updating token representations via Multi-Head Self-Attention. For layer *l* ($l<N_{s}$), given input $Z^{\left(l-1\right)}\in R^{B\times (L_{l}+1)\times d_{\text{model}}}$, the query, key, and value matrices are computed:


(6) $$\begin{array}{c} Q=Z^{\left(l-1\right)}W_{q}^{l},\\ K=Z^{\left(l-1\right)}W_{k}^{l},\\ V=Z^{\left(l-1\right)}W_{v}^{l} \end{array}$$

with $W_{q}^{l},W_{k}^{l},W_{v}^{l}\in R^{d_{\text{model}}\times d_{\text{model}}}$. The attention matrix is:


(7) $$A^{l}=\text{Softmax}\left(\frac{QK^{T}}{\sqrt{d_{k}}}\right)\in R^{B\times h\times (L_{l}+1)\times (L_{l}+1)}$$

where $d_{k}$ is the dimension per attention head, calculated as $d_{k}=d_{\text{model}}/h$. The subsequent $N_{a}=3$ layers integrate the ATS mechanism, designed to dynamically focus on informative regions.

#### 2.4.1 Importance scoring based on attention matrix

In ATS layers, token importance is derived from the [CLS] token’s attention in the multi-head self-attention matrix $A^{l}$. The first row of $A^{l}$ captures interactions between [CLS] and all input tokens. To stabilize estimation, attention matrices from all heads are averaged:


(8) $${\bar{A}}^{l}=\frac{1}{h}\sum_{i=1}^{h}A_{i}^{l}\in R^{B\times (L_{l}+1)\times (L_{l}+1)}$$

where *h* is the number of heads, *B* is batch size, and $L_{l}$ is the number of non-[CLS] tokens.

The [CLS] attention vector ${\bar{A}}^{l}[:,0,:]$ indicates each token’s relative contribution. The importance score of token *j* is:


(9) $$s_{j}^{l}={\bar{A}}^{l}[:,0,j],\;j=1,\ldots ,L_{l}$$

forming $s^{l}\in R^{B\times L_{l}}$. Normalizing the importance scores along the token dimension produces the sampling probability distribution:


(10) $$p_{b,j}^{l}=\frac{s_{b,j}^{l}}{\sum_{r=1}^{L_{l}}s_{b,r}^{l}}$$

#### 2.4.2 Adaptive token sampling strategy

**Figure 2:**
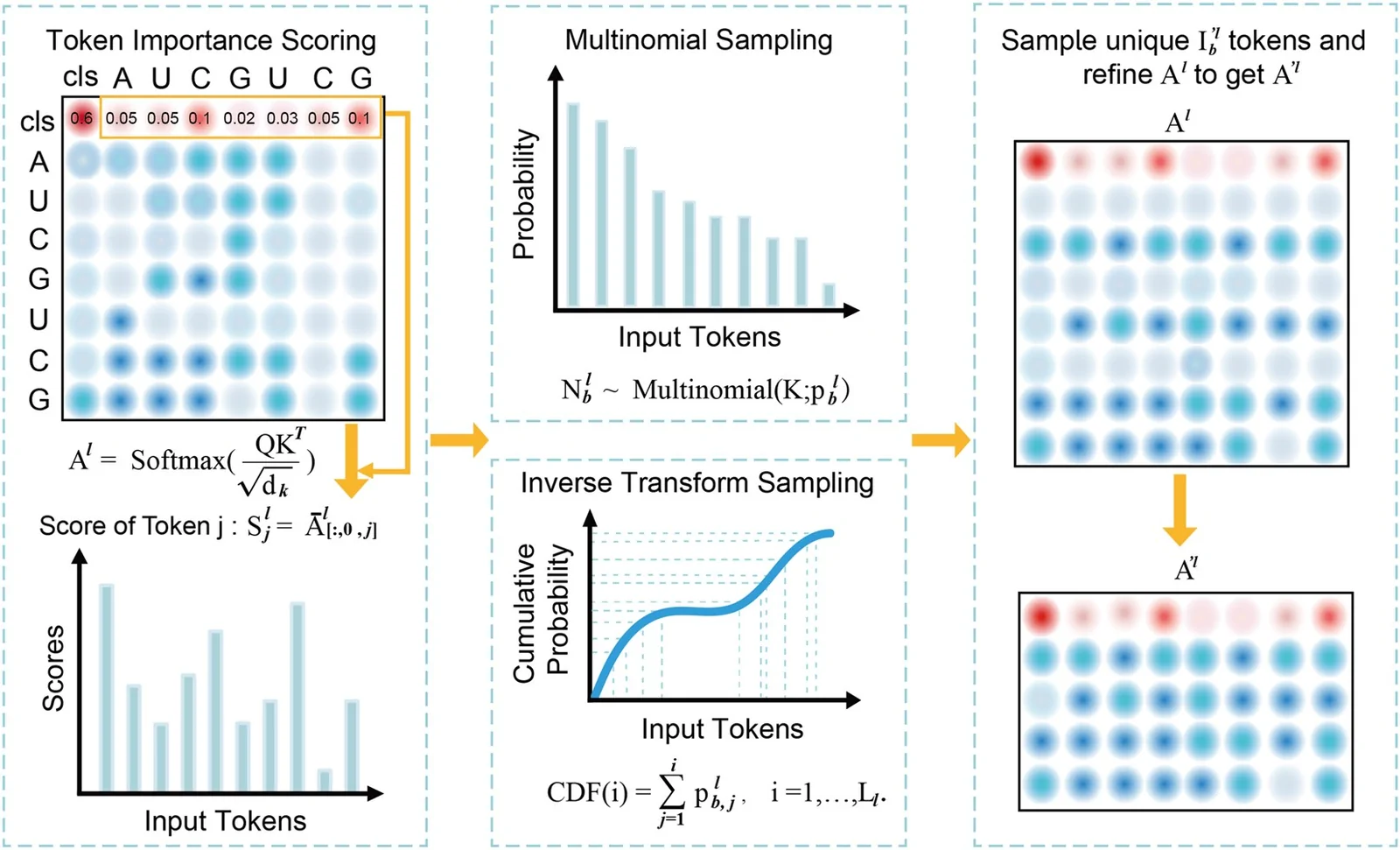
Mechanism of adaptive token sampling. Left: attention-based token importance scoring. Middle: multinomial and inverse transform sampling. Right: index deduplication and sorting to construct the pruned token representation.

After computing the importance distribution $p^{l}$, a simple strategy is to prune tokens using a fixed criterion, such as retaining a predefined proportion at each layer. However, genomic sequences exhibit highly variable distributions of informative regions, where either many or only a few bases may be functionally relevant. This variability motivates the use of an adaptive token sampling strategy. Based on the normalized importance distribution $p^{l}$, we apply two probabilistic sampling methods to select informative tokens, as illustrated in Fig. 2.

### 2.5 Multinomial sampling

Because the importance scores $s_{j}$ are normalized to form the sampling probabilities, the model can draw *K* samples from this distribution according to a multinomial random variable *N*: $N_{b}^{l}∼\text{Multinomial}(K;p_{b}^{l})$.

where *K* denotes the target number of sampled tokens. Duplicate samples are collapsed into unique indices. As a result, the final number of retained tokens can range from *K* down to one in extreme cases where a single token dominates the importance distribution. This strategy naturally favours high-importance tokens while preserving diversity through probabilistic selection.

### 2.6 Inverse transform sampling

In contrast to multinomial sampling, inverse transform sampling selects tokens based on the cumulative distribution of importance scores. Given the discrete probability distribution $p_{b}^{l}$, the cumulative distribution function (CDF) is defined as


(11) $$\text{CDF}(i)=\sum_{j=1}^{i}p_{b,j}^{l},\;i=1,\ldots ,L_{l}$$

Following the inverse transform sampling principle, *K* independent samples $u_{1},\ldots ,u_{K}∼U[0,1]$ are drawn, and the corresponding token indices are obtained by


(12) $$i_{k}=\text{min}\left\{i:\text{CDF}(i)\geq u_{k}\right\}$$

After sampling, index post-processing is applied: sampled non-[CLS] indices are shifted by +1 to correct positional offsets, merged with the fixed [CLS] index, and deduplicated and sorted:


(13) $$I_{b}^{\prime l}=\text{sort}\left(\text{unique}\left(\left\{0\}\cup (I_{b}^{l}+1\right)\right)\right)$$

This mechanism favours tokens with higher importance scores while preserving probabilistic diversity. Compared with deterministic top-*K* selection, it better preserves the original importance distribution, especially in early layers with relatively uniform attention weights.

#### 2.6.1 Token representation pruning

After each ATS-enabled layer, the retained token representations are selected from the layer output using the index set in Eq. 13:


(14) $${\tilde{Z}}_{b}^{l}=Z_{b}^{l}[I_{b}^{\prime l},:]\in R^{|I_{b}^{\prime l}|\times d_{\text{model}}}$$

After batch padding and masking, the retained representations are passed to the next Transformer layer:


(15) $$Z^{\left(l+1\right)}={\text{EncoderLayer}}_{l+1}\left({\tilde{Z}}^{l};M^{l}\right)$$

During backpropagation, gradients propagate through the retained token representations and Transformer computations along the realized selection path, but not through the discrete selection indices. Thus, the sampling strategies introduce no additional trainable parameters. After processing through all *N* Transformer layers, the final representation of the classification token ([CLS]) is extracted:


(16) $$z_{\text{cls}}^{N}=Z^{N}[:,0,:]\in R^{B\times d_{\text{model}}}$$

### 2.7 Data construction

A precise and reliable dataset is fundamental to effective research. Here, we constructed a benchmark dataset based on RMVar 2.0 (Huang *et al*. 2025), which offers comprehensive information on RNA modification–associated single-nucleotide polymorphisms (RNAm-SNPs), expanding coverage and biological relevance compared with RMBase v3.0 (Xuan *et al*. 2024). The extracted sequences were obtained directly from the human reference genome (GRCh38). Using genomic coordinates of annotated human modification sites, we extracted positive-strand sequences centred on A-to-I, m1A, m5C, and m7G modifications, extending 500 nucleotides upstream and downstream to form sequences of 1001 nucleotides. Redundancy was reduced with CD-HIT (Fu *et al*. 2012) at a 90% similarity threshold, resulting in 37 397, 5165, 11 377, and 8774 positive samples for the four modifications, respectively. Equal numbers of negative samples were randomly selected from the human genome, ensuring no overlap with positives. Given the novelty of this dataset, all methods were evaluated using both five-fold cross-validation and an independent data split. The independent split used an 8:1:1 ratio for training, validation, and testing. For five-fold cross-validation, four folds were used for training and the remaining fold for validation and evaluation, with results reported as the mean and standard deviation across folds.

### 2.8 Evaluation metrics

To comprehensively evaluate the performance of our model in predicting RNA modification sites, we adopted five commonly used evaluation metrics: accuracy (ACC), sensitivity (Se), specificity (Sp), Matthews correlation coefficient (MCC), and area under the receiver operating characteristic curve (AUC) (Sun *et al*. 2019, Zou *et al*. 2023, Zhao *et al*. 2024, Yu *et al*. 2025, Li *et al*. 2026). The definitions and properties of these metrics are as follows:


(17) $$\text{ACC}=\frac{TP+TN}{TP+FP+FN+TN}$$

(18) $$\text{Se}=\frac{TP}{TP+FN},\;\text{Sp}=\frac{TN}{TN+FP}$$

(19) $$\text{MCC}=\frac{TP\times TN-FP\times FN}{\sqrt{\left(TP+FP)(TP+FN)(TN+FP)(TN+FN\right)}}$$

where TP, TN, FP, and FN represent the numbers of true positives, true negatives, false positives, and false negatives, respectively. The range of MCC is from -1 to 1, where 1 indicates perfect prediction, 0 represents random prediction, and -1 indicates completely incorrect prediction. The range of AUC is from 0 to 1, with higher values indicating better performance, and 0.5 representing no discriminatory ability.

To further assess the computational efficiency of the proposed model, we evaluated its computational cost using two commonly adopted indicators: floating-point operations (FLOPs) (Chen *et al*. 2023) and the number of trainable parameters. FLOPs measure the total amount of computation required for a single forward pass, reflecting the model’s inference complexity, while the parameter count indicates the model’s memory footprint and storage requirements.

### 2.9 Implementation details

All experiments were implemented in PyTorch and conducted on a single NVIDIA GeForce RTX 3090 GPU, with a fixed random seed used throughout. ATSFormer was trained using the Adam optimizer with a learning rate of 0.001 and a batch size of 64. Training was performed for up to 100 epochs with early stopping based on validation accuracy, using a patience of 10 epochs. Standard two-class cross-entropy loss was used, with the classification head producing two logits per sample. For a fair comparison, all baseline methods used the same data partitions and early-stopping strategy as ATSFormer. The best-performing hyperparameters and input sequence lengths reported in their original studies were adopted, while unspecified settings were aligned with ATSFormer. Subsequent efficiency, ablation, and interpretability analyses were all conducted on the A-to-I dataset. Detailed hyperparameter configurations and training settings are summarized in Table S2, available as supplementary data at *Bioinformatics* online.

## 3 Results

### 3.1 Evaluation on benchmark datasets

We systematically compared ATSFormer with several representative biological sequence foundation models, including ERNIE-RNA, Nucleotide Transformer, and GROVER, as well as RNA modification site prediction methods, including RDDSVM, PreAIS, iMRM, MRM-BERT, and MultiRM, on four biologically meaningful and challenging benchmark datasets: A-to-I, m1A, m5C, and m7G. To ensure a fair comparison, each competing method was evaluated using the input sequence length recommended in its original study when available, while the optimal sequence length identified in this study, 501 nt, was used for methods without a specified input length. These datasets provide a rigorous benchmark for assessing whether advanced tokenization strategies can effectively model long genomic sequences. Model performance was assessed under both five-fold cross-validation and independent data split settings, with the results summarized in Table 1 and detailed in Tables S3 and S4, available as supplementary data at *Bioinformatics* online.

**Table 1:** Performance comparison on four benchmark datasets.

Data splitting strategies		Five-fold cross-validation			Independent data splits		
Dataset	Methods	ACC (%)	MCC (%)	AUC (%)	ACC (%)	MCC (%)	AUC (%)
A-to-I	ATSFormer (501)	**92.61**	**85.28**	**96.89**	**92.79**	**85.68**	**97.16**
	ERNIE-RNA (501)	87.67	75.82	93.57	89.04	78.09	93.48
	Nucleotide Transformer (501)	74.17	48.62	80.69	73.81	47.76	80.00
	GROVER (501)	89.04	78.11	93.28	88.14	76.34	93.03
	iMRM (41)	89.90	79.80	95.50	89.97	79.95	95.68
	RDDSVM (101)	87.19	74.39	91.31	87.73	75.45	91.70
	PreAIS (101)	87.22	74.46	92.49	87.54	75.09	92.82
	MRM-BERT (101)	90.00	80.16	94.41	90.76	81.53	94.45
	MultiRM (51)	79.56	59.11	86.53	81.48	63.17	87.62
m1A	ATSFormer (501)	**86.95**	**73.97**	**94.81**	**85.62**	**71.25**	**94.18**
	ERNIE-RNA (501)	84.43	69.04	91.85	83.88	68.12	91.52
	Nucleotide Transformer (501)	75.80	51.75	83.30	72.88	45.96	80.87
	GROVER (501)	77.82	56.08	85.80	75.10	50.47	83.40
	iMRM (41)	86.45	72.92	94.45	84.65	69.31	92.50
	RDDSVM (101)	79.97	60.84	87.83	80.50	62.27	88.54
	PreAIS (101)	79.62	59.32	87.67	80.41	61.04	88.55
	MRM-BERT (101)	81.85	63.75	88.18	66.31	33.43	71.56
	MultiRM (51)	77.39	54.89	84.27	52.78	6.49	60.87
m5C	ATSFormer (501)	**89.09**	**78.31**	**95.94**	**89.50**	**79.03**	**95.94**
	ERNIE-RNA (501)	86.74	73.94	94.37	86.56	73.17	94.27
	Nucleotide Transformer (501)	79.32	58.71	87.13	77.64	55.27	85.50
	GROVER (501)	80.81	61.82	88.80	78.73	57.58	87.31
	iMRM (41)	88.40	76.85	95.64	85.59	71.34	94.05
	RDDSVM (101)	82.96	65.94	90.49	82.25	64.52	90.08
	PreAIS (101)	82.47	64.95	90.62	82.21	64.50	90.60
	MRM-BERT (101)	75.84	53.66	87.35	71.22	42.94	78.18
	MultiRM (51)	80.60	61.20	88.57	56.88	14.09	59.04
m7G	ATSFormer (501)	**85.72**	**71.65**	**93.59**	**86.06**	**72.41**	**93.45**
	ERNIE-RNA (501)	80.91	62.72	89.30	80.49	62.22	88.97
	Nucleotide Transformer (501)	73.09	46.26	80.56	70.82	42.22	78.29
	GROVER (501)	77.65	55.38	85.26	75.65	51.31	83.70
	iMRM (41)	83.42	67.03	91.92	85.15	70.70	92.70
	RDDSVM (101)	77.11	54.30	84.97	76.79	53.59	84.89
	PreAIS (101)	77.89	55.80	85.73	77.19	54.48	85.37
	MRM-BERT (101)	84.79	70.35	92.10	66.04	32.24	72.32
	MultiRM (51)	75.49	50.95	82.81	66.16	32.68	71.97

Best results are shown in bold, and second-best results are underlined.

**Figure 3:**
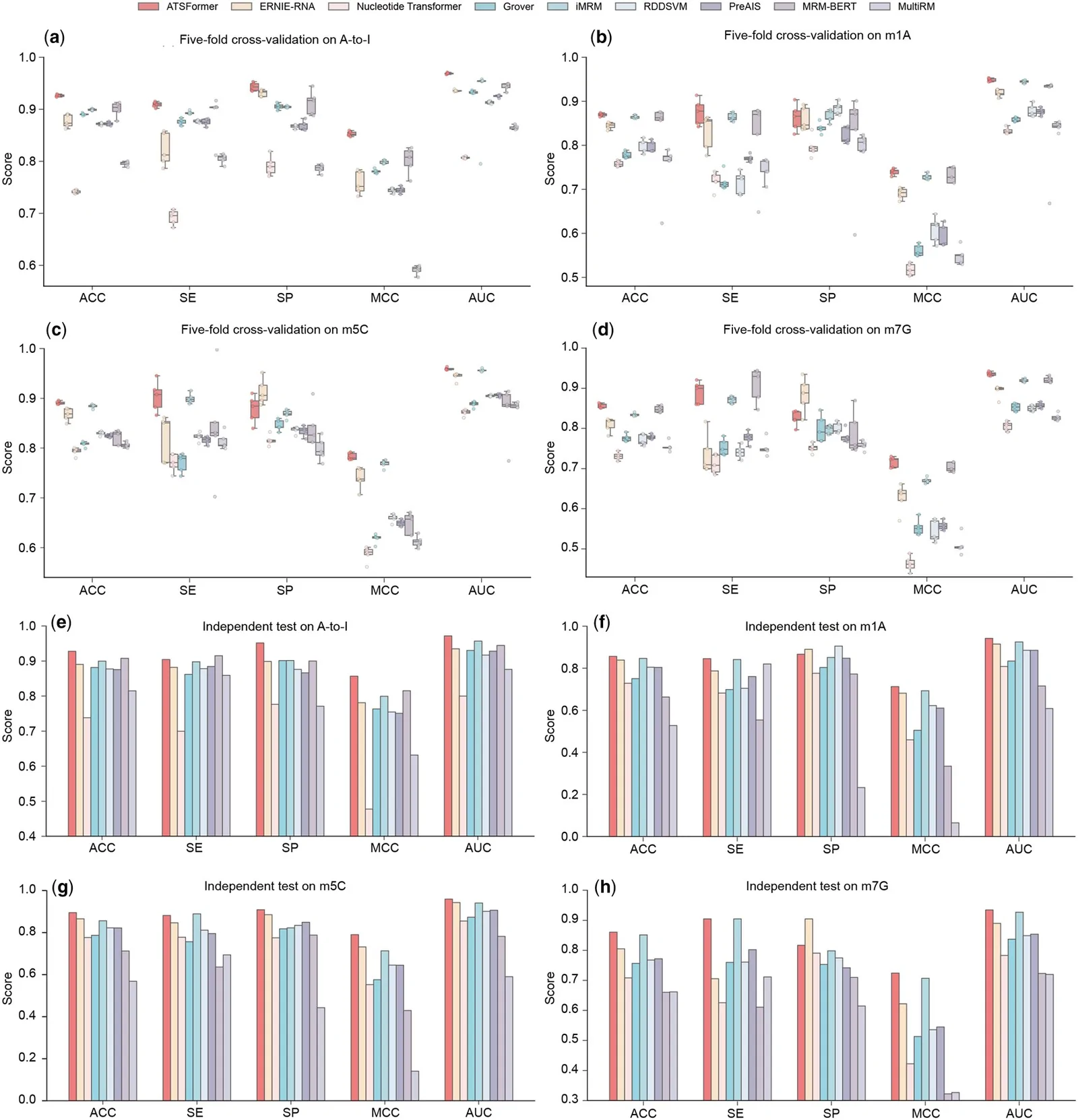
Performance comparison on four benchmark datasets. (a–d) Boxplots showing model performance under five-fold cross-validation on the A-to-I, m1A, m5C, and m7G datasets, respectively. (e–h) Bar plots showing model performance under independent data split settings on the A-to-I, m1A, m5C, and m7G datasets, respectively.

First, to reduce the randomness introduced by data partitioning, we evaluated model performance and stability under the five-fold cross-validation protocol. ATSFormer consistently achieved the best overall performance across all four datasets (Fig. 3a–d), outperforming competing methods on key metrics including ACC, MCC, and AUC. Notably, on the most challenging A-to-I dataset, ATSFormer achieved an ACC of 92.61%, an MCC of 85.28%, and an AUC of 96.89%, representing substantial improvements of 2.61%, 5.12%, and 2.48%, respectively, over the second-best model, MRM-BERT. These results indicate that ATSFormer is more effective at discriminating positive and negative samples in the context of complex RNA-editing mechanisms. On the m1A, m5C, and m7G datasets, ATSFormer also maintained leading performance in terms of ACC, AUC, and MCC, while achieving a more balanced trade-off between Se and Sp.

To further assess the generalization ability of ATSFormer, we conducted additional experiments under the independent data split setting. ATSFormer consistently outperformed all competing methods across the four datasets (Fig. 3e–h), achieving ACC values of 92.79%, 85.62%, 89.50%, and 86.06% on the A-to-I, m1A, m5C, and m7G datasets, respectively. Moreover, ATSFormer maintained superior performance in terms of ACC, MCC, and AUC under both evaluation protocols, demonstrating strong robustness and stability. In contrast, several baseline methods exhibited pronounced performance degradation under independent data splits, suggesting potential overfitting or limited generalization capability. Taken together, these results demonstrate that ATSFormer achieves substantial and consistent advantages over existing competing methods.

### 3.2 Effects of sampling configuration on model efficiency

**Figure 4:**
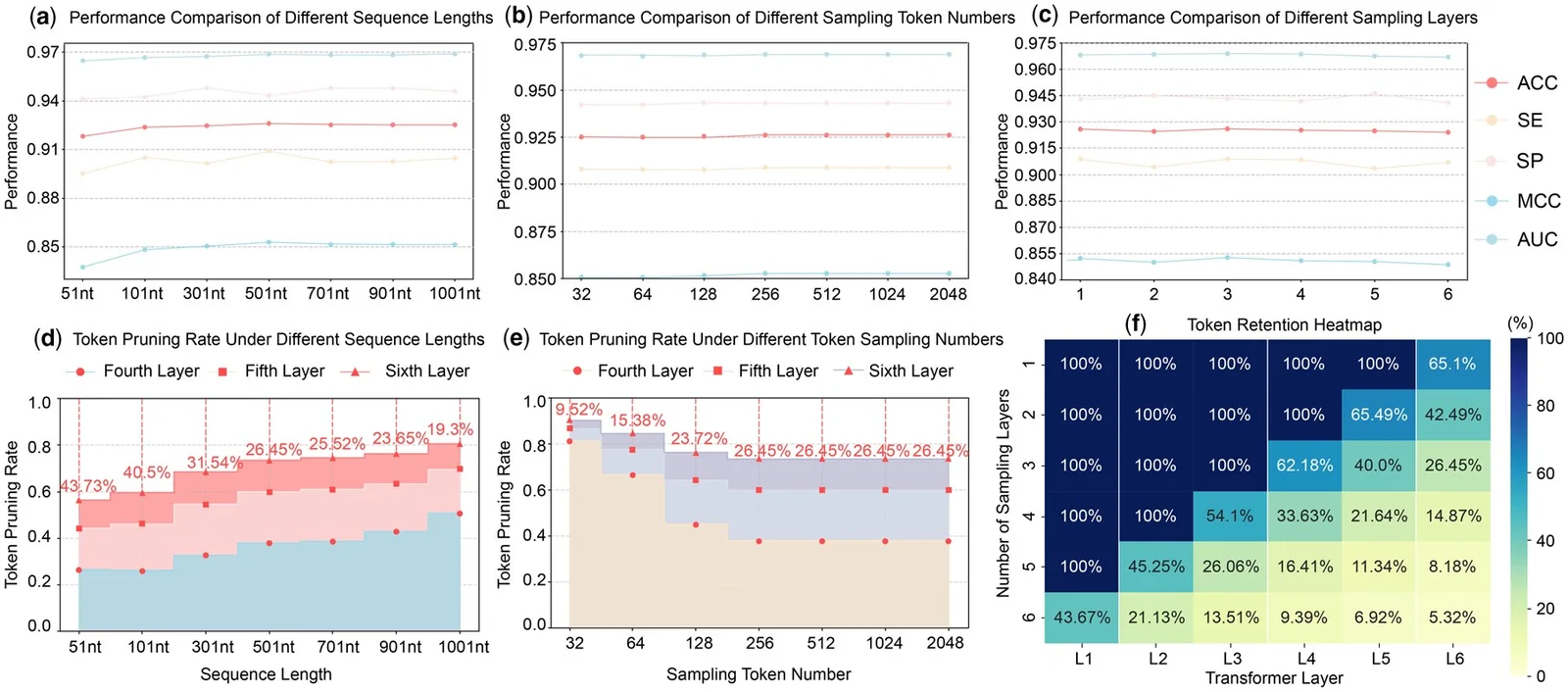
Performance and efficiency comparison across different sequence lengths and adaptive sampling strategies. (a–c) Performance comparison under different input sequence lengths, numbers of sampling tokens, and numbers of sampling layers. The five coloured lines represent ACC, sensitivity (Se), specificity (Sp), MCC, and AUC. (d–e) Token pruning rates under different input sequence lengths and numbers of sampling tokens. (f) Token retention heatmap showing the proportion of tokens retained at each Transformer layer when different numbers of sampling layers are applied. Rows indicate the number of Transformer layers equipped with adaptive token sampling, columns indicate Transformer layers L1–L6, and colours represent the percentage of retained tokens.

We systematically analysed the effects of input sequence length, the number of sampled tokens, and the number of Transformer sampling layers on model performance and computational efficiency. The model achieved optimal performance with an input sequence length of 501 nt (Fig. 4a). As the sequence length increased, the pruning rate also increased (Fig. 4d), suggesting that ATSFormer can adaptively remove redundant tokens from longer sequences.

To further evaluate its scalability, we extended the input sequence length on the A-to-I dataset from the best-performing setting of 501 nt (ACC: 0.9279) to 2001 nt (ACC: 0.9233) and 3001 nt (ACC: 0.9213). Although the ACC decreased by less than 0.7 percentage points, the performance remained comparable to that of the corresponding w/o ATS settings (2001 nt ACC: 0.9224; 3001 nt ACC: 0.9249). Meanwhile, the token retention ratio decreased from 26.45% to 14.26% and 7.12%, respectively, indicating that the adaptive sampling mechanism effectively discards tokens with minimal predictive contribution while retaining key tokens in central or high-information regions. These results demonstrate an effective balance between information compression and feature preservation and suggest that the computational advantage of ATSFormer becomes more pronounced as the genomic context increases. They support the potential application of ATSFormer to large-scale genomic analyses in which successive genomic regions are processed separately.

Analysis of the number of sampling tokens shows that performance improves as more tokens are retained (Fig. 4b). Performance peaks when 256 sampling tokens are kept, and further increases provide no additional gain while the pruning rate remains largely unchanged, suggesting that task-relevant information is already captured (Fig. 4e).

For Transformer sampling layers, systematic experiments were conducted on a six-layer architecture. When adaptive sampling is applied only to the last three layers, the model achieves the best trade-off between performance and efficiency, retaining about 26.45% of tokens and substantially reducing computational cost (Fig. 4c). When all six layers adopt adaptive sampling, accuracy decreases by only 0.15% compared with the unsampled configuration, indicating minimal risk of performance degradation. Only tokens with low predictive contribution are pruned. In this setting, only 5.32% of tokens are retained, greatly shortening effective sequence length, lowering memory usage, and reducing computational burden, thereby accelerating training and inference (Fig. 4f).

As shown in Table 2, ATSFormer requires substantially fewer FLOPs and parameters than existing state-of-the-art Transformer-based models. Specifically, the FLOPs of these competing models are approximately 96–1459 times higher than those of ATSFormer. For a single sample, ATSFormer requires only 31.395M FLOPs, and the ATS module reduces FLOPs by 12.3% without introducing any additional parameters into the backbone model. These results demonstrate that the proposed method effectively reduces computational complexity while maintaining high predictive accuracy, thereby achieving a favourable balance between efficiency and performance.

**Table 2:** Comparison of FLOPs and number of parameters across existing transformer-based methods.

Methods	Single-sample FLOPs	All-sample FLOPs	Params
ATSFormer-M	31.395 M	7.349 T	0.215 M
w/o ATS	35.785 M	8.377 T	0.215 M
ATSFormer-IT	32.022 M	7.496 T	0.215 M
Nucleotide Transformer	41 674.024 M	9755.222 T	479.486 M
GROVER	3012.719 M	705.229 T	86.314 M
ERNIE-RNA	29 283.084 M	6854.701 T	59.005 M
MRM-BERT	45 798 M	10 720.593 T	228.411 M

### 3.3 Ablation study

To comprehensively evaluate the effectiveness of ATSFormer, we conducted ablation experiments to assess the contribution of each model component. Specifically, we constructed five ablated variants by selectively removing key modules: Adaptive Token Sampling (w/o ATS), the entire Word-Detection Module with both CNN layers removed (w/o CNN), only the second CNN layer removed (w/o CNNoneLayer), the Adaptive Token Sampling Transformer Module removed (w/o Transformer), and the Positional-Aware Token Embedding removed (w/o PositionalEncoding).

**Figure 5:**
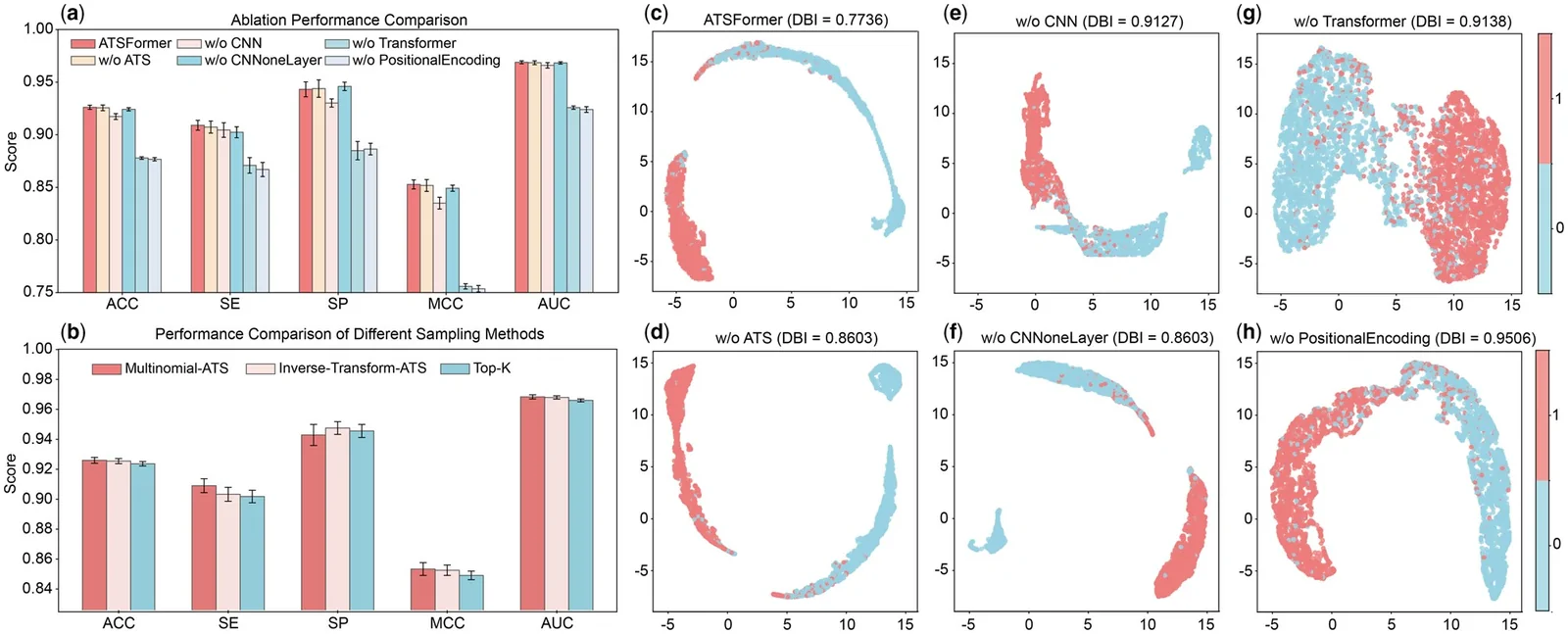
Performance comparison in ablation study. (a) Performance comparison of ATSFormer and ablation models. (b) Performance comparison of different sampling methods. (c–h) UMAP analysis of ATSFormer and ablation models.

As shown in Fig. 5a, removing any component results in a clear performance degradation, indicating that all modules contribute to the overall effectiveness of ATSFormer. In particular, excluding the Adaptive Token Sampling Transformer or the Positional-Aware Token Embedding leads to notable accuracy drops of 4.82% and 4.94%, respectively, underscoring their critical roles. The Word-Detection Module further enhances performance by improving local feature extraction, while the ATS mechanism effectively reduces the computational cost of token sampling without compromising model expressiveness. We further applied Uniform Manifold Approximation and Projection (UMAP) (Healy and Mcinnes 2024) to visualize learned representations and quantified clustering quality using the Davies–Bouldin Index (Singh *et al*. 2020). The full ATSFormer achieved the lowest DBI, indicating superior feature separability (Fig. 5c–h).

In addition, we compared three token sampling strategies, including Multinomial-ATS, Inverse-Transform-ATS, and Top-K sampling (Fig. 5b). Both adaptive sampling methods consistently outperformed Top-K sampling, which suffers from rigid thresholding that may discard informative tokens, particularly during early training stages.

### 3.4 Structural insights into motif function

ATSFormer leverages in silico saturation mutagenesis (ISM) (Nair *et al*. 2022), a technique that evaluates the impact of individual nucleotide positions on model predictions by simulating mutations, to identify key sequence motifs associated with A-to-I editing sites (Fig. S2, available as supplementary data at *Bioinformatics* online). The natural next step is to investigate whether these motifs occur in structural contexts relevant to ADAR-mediated site-specific RNA editing. Previous studies have demonstrated that members of the ADAR family, specifically the catalytically active ADAR1 isoforms (ADAR1 p110 and ADAR1 p150) and ADAR2 isoforms (ADAR2a and ADAR2b), recognize and bind double-stranded RNA regions, thereby catalysing the deamination of adenosine to inosine (George *et al*. 2011, Filippini *et al*. 2018).

**Figure 6:**
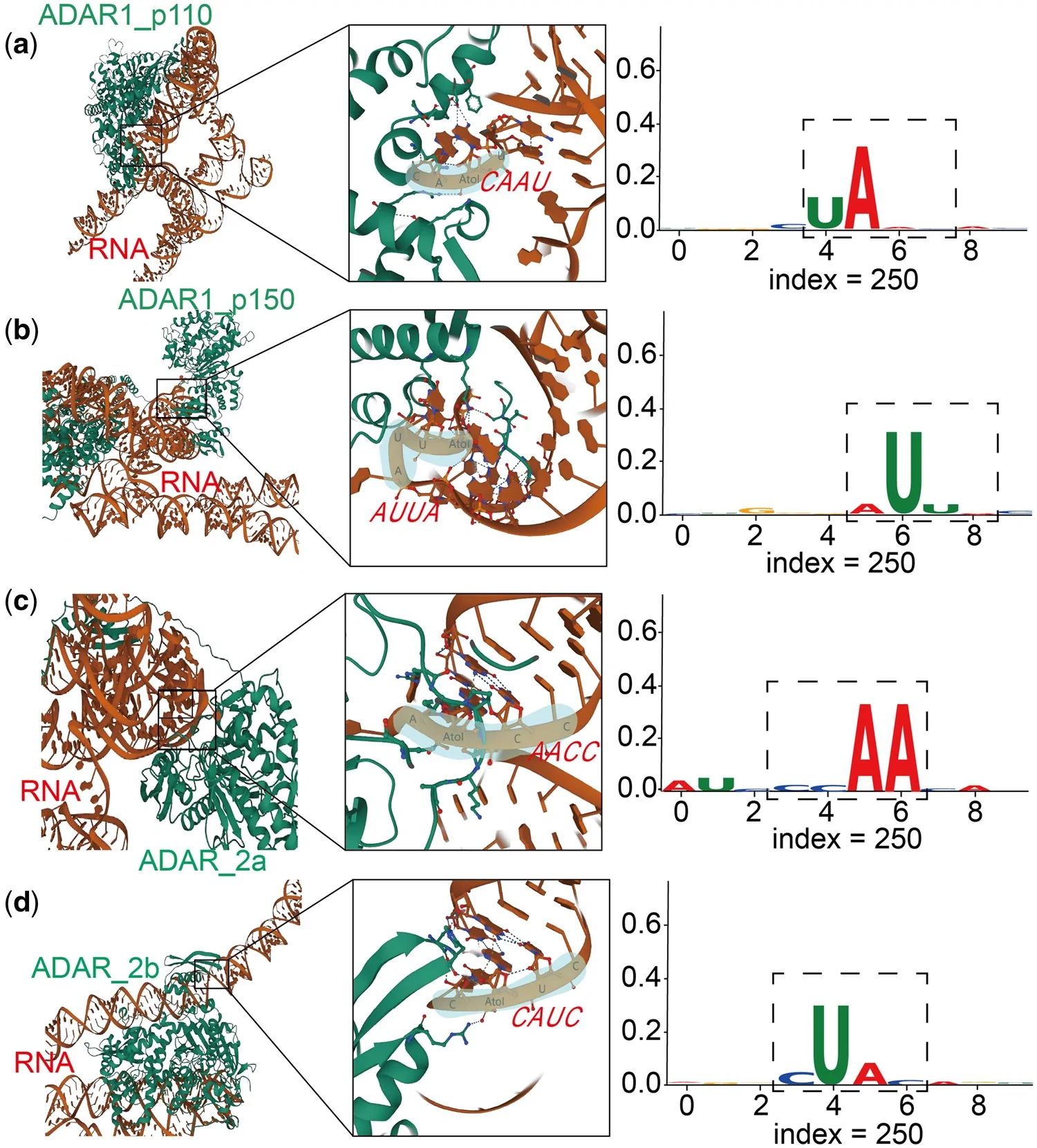
Structural analysis of ADAR–RNA interactions. Four RNA sequences were randomly selected from the dataset, and AlphaFold3 was used to predict their interactions with ADAR1 p110 (a), ADAR1 p150 (b), ADAR2a (c), and ADAR2b (d). RNA molecules are highlighted in orange and ADAR proteins in cyan, with RNA-binding sites indicated by blue circles. Corresponding motifs identified by our model are shown on the right side of each panel, with black boxes emphasizing their spatial correspondence to the predicted binding sites.

To assess this spatial consistency, we employed AlphaFold3 (Abramson *et al*. 2024) to model potential interactions between these four catalytically active ADAR isoforms and RNA sequences containing annotated editing sites. Notably, the predicted structural complexes suggest spatial correspondence between the RNA-binding interfaces of the ADAR enzymes and the motif regions identified through the ISM-based analysis. The motif region in Fig. 6a corresponds to the AAU context, which is among the preferred ADAR2 triplets, whereas those in Fig. 6c and d correspond to the moderately preferred AAC and CAU contexts, respectively (Lehmann and Bass 2000). The U–A adjacency in Fig. 6b is also consistent with the strong preference of ADAR enzymes for uridine immediately 5${\;}^{\prime }$ to the edited adenosine (Reautschnig *et al*. 2025).

We further observed that the annotated A-to-I editing sites are located within RNA regions capable of forming specific secondary structures, including canonical double-stranded RNA (dsRNA) regions (Fig. 6b–d) or a local bulge structure (Fig. 6a) (Zambrano-Mila *et al*. 2023, Sun *et al*. 2026). Overall, the spatial correspondence between the predicted ADAR–RNA interaction regions and the identified motifs supports the view that their sequence and structural features may contribute to site-selective A-to-I RNA editing.

## 4 Conclusion

This study demonstrates that adaptive token pruning is a powerful strategy for addressing the scalability limitations of Transformer-based genomic models. By integrating attention-guided sampling directly into the model architecture, ATSFormer effectively concentrates computation on biologically informative regions while preserving interpretability and performance. Unlike fixed-length or deterministic pruning approaches, the proposed adaptive sampling mechanism flexibly accommodates heterogeneous genomic signal distributions. The strong performance across multiple RNA modification tasks, together with the substantial computational savings and increasing token compression observed for longer sequences, demonstrates the potential of ATSFormer for large-scale genomic analyses. Although the present study did not perform an end-to-end scan of the complete human genome, genome-scale analysis can be conducted by successively processing genomic regions or candidate loci. These findings open up new possibilities for extending this framework to whole-genome modelling and other regulatory genomics applications.

## Supplementary Material

btag621_Supplementary_Data

## Data Availability

All of the source data and code are freely available at GitHub (https://github.com/1gao2/ATSFormer) and Zenodo (https://doi.org/10.5281/zenodo.21813541).
