# Supplementary material for: A token-pruning framework enables efficient representation of the human genome for RNA modification analysis
Source: Bioinformatics. 2026 Sep 1;42(9):btag621. doi: 10.1093/bioinformatics/btag621 (PMC13553080; doi:10.1093/bioinformatics/btag621)
Supplement: Supplementary file 1 [file btag621_supplementary_data.docx]

**Supplementary Information**

**1 Implementation Details**

**1.1 Benchmark Datasets**

To facilitate reproducibility and provide a more detailed characterization of the benchmark datasets used in this study, we summarize the composition and partitioning of the four RNA modification datasets in Table 1. All datasets were constructed by systematically processing and curating data derived from the RMVar 2.0 database, which provides comprehensive annotations of RNA modification–associated single-nucleotide variants in the human genome. The benchmark datasets include adenosine-to-inosine (A-to-I) RNA editing, 1-methyladenosine (m1A), 5-methylcytidine (m5C), and 7-methylguanosine (m7G), corresponding to original bases A, A, C, and G, respectively. For each modification type, genomic coordinates of experimentally supported modification sites were extracted from RMVar 2.0, and positive samples were constructed by retrieving 1,001-nucleotide sequences centered on each modification site.

To reduce redundancy and potential sequence bias, CD-HIT was applied with a 90% sequence identity threshold. After filtering, the final datasets contained 74,794 (A-to-I), 10,330 (m1A), 22,754 (m5C), and 17,548 (m7G) samples in total, including both positive and negative instances. Negative samples were randomly selected from the human genome while ensuring no overlap with known modification sites reported in RMVar 2.0, resulting in balanced datasets with equal numbers of positive and negative samples for each modification type. For independent evaluation, each dataset was further divided into training, validation, and test sets using an approximate 8:1:1 ratio. Specifically, for the A-to-I dataset, 29,917 positive and 29,917 negative samples were allocated to the training set, while 3,740 samples per class were assigned to both the validation and test sets. Similar partitioning strategies were applied to the m1A, m5C, and m7G datasets, as detailed in Supplementary Table 1.

**Supplementary Table 1: Overview of the datasets for the four types of RNA modifications.**

| Types | Full name | Original base | Number of samples | | | |
| --- | --- | --- | --- | --- | --- | --- |
|  |  |  | Training | Validation | Test | Total |
| A-to-I | Adenosine-to-inosine RNA editing | A | 59,834 | 7,480 | 7,480 | 74,794 |
| m1A | 1-methyladenosine | A | 8,260 | 1,034 | 1,036 | 10,330 |
| m5C | 5-methylcytidine | C | 18,202 | 2,276 | 2,276 | 22,754 |
| m7G | 7-methylguanosine | G | 14,036 | 1,756 | 1,756 | 17,548 |

**1.2 Training and Inference Details**

The proposed model was implemented in PyTorch and trained on a single NVIDIA GPU. Input RNA sequences were encoded using a 1-mer scheme and processed by a CNN–Transformer architecture with Adaptive Token Sampling (ATS). Model training was carried out using the Adam optimizer with cross-entropy loss, and early stopping was applied based on validation accuracy. Comparative experiments were conducted under two training strategies: five-fold cross-validation and an independent train–validation–test split, whereas all other experiments were performed exclusively under the five-fold cross-validation setting to ensure robust and fair evaluation. Detailed hyperparameter configurations and training settings are summarized in Supplementary Table 2.

**Supplementary Table 2: Hyperparameter settings of ATSFormer.**

| Category | Hyperparameter | Value | Description |
| --- | --- | --- | --- |
| Input & Encoding | Sequence length | 501 | Length of input RNA sequence |
|  | Vocabulary size | 5 | {A, T, C, G, –} |
|  | k-mer size | 1 | 1-mer encoding |
| CNN Module | Number of CNN layers | 2 | Two stacked 1D convolution layers |
|  | Kernel number | 128 | Number of convolution filters |
|  | Kernel size (layer 1) | 7 | First convolution kernel size |
|  | Stride (layer 1) | 3 | Stride of first convolution |
|  | Kernel size (layer 2) | 3 | Second convolution kernel size |
|  | Stride (layer 2) | 1 | Stride of second convolution |
| Embedding | Embedding dimension | 64 | Transformer hidden dimension |
| Transformer Encoder | Number of layers | 6 | Total Transformer encoder layers |
|  | Attention heads | 8 | Multi-head self-attention heads |
|  | FFN hidden dimension | 64 | Feed-forward network hidden size |
|  | Dropout rate | 0.1 | Dropout probability |
| Adaptive Token Sampling | Sampling start layer | 4 | ATS applied after the 3rd layer |
|  | Maximum kept tokens | 256 | Upper bound of sampled tokens |
| Classifier | Hidden dimension | 16 | Hidden size of classification head |
|  | Output classes | 2 | Binary classification |
| Training | Optimizer | Adam | Optimization algorithm |
|  | Learning rate | 0.001 | Initial learning rate |
|  | Batch size | 64 | Mini-batch size |
|  | Epochs | 100 | Maximum training epochs |
|  | Loss function | CrossEntropyLoss | Classification loss |
|  | Early stopping patience | 10 | Epochs without improvement |

**2 Additional Experimental Results**

**2.1 Evaluation on Benchmark Datasets**

More comprehensive comparison results under the five-fold cross-validation and independent train–validation–test split settings are reported in Supplementary Table 3 and Supplementary Table 4, respectively.

**Supplementary Table 3. Performance comparison under five-fold cross-validation on four benchmark datasets.**

| Dataset | Methods | ACC(%) | Se(%) | Sp(%) | MCC(%) | AUC(%) |
| --- | --- | --- | --- | --- | --- | --- |
| A-to-I | ATSFormer (501) | **92.61 ± 0.20** | **90.90 ± 0.47** | **94.32 ± 0.71** | **85.28 ± 0.43** | **96.89 ± 0.14** |
|  | ERNIE-RNA (501) | 87.67 ± 1.13 | 82.23 ± 2.88 | 93.10 ± 0.57 | 75.82 ± 2.02 | 93.57 ± 0.15 |
|  | Nucleotide Transformer (501) | 74.17 ± 0.28 | 69.23 ± 1.29 | 79.13 ± 1.67 | 48.62 ± 0.68 | 80.69 ± 0.17 |
|  | Grover (501) | 89.04 ± 0.17 | 87.55 ± 0.54 | 90.53 ± 0.52 | 78.11 ± 0.34 | 93.28 ± 0.22 |
|  | iMRM (41) | 89.90 ± 0.15 | 89.35 ± 0.27 | 90.45 ± 0.15 | 79.80 ± 0.29 | 95.50 ± 0.14 |
|  | RDDSVM (101) | 87.19 ± 0.20 | 87.69 ± 0.21 | 86.69 ± 0.32 | 74.39 ± 0.40 | 91.31 ± 0.22 |
|  | PreAIS (101) | 87.22 ± 0.29 | 87.50 ± 0.59 | 86.95 ± 0.81 | 74.46 ± 0.57 | 92.49 ± 0.21 |
|  | MRM-BERT (101) | 90.00 ± 1.27 | 88.79 ± 3.84 | 91.24 ± 2.09 | 80.16 ± 2.29 | 94.41 ± 0.65 |
|  | MultiRM (51) | 79.56 ± 0.40 | 80.49 ± 0.84 | 78.60 ± 0.72 | 59.11 ± 0.78 | 86.53 ± 0.36 |
| m1A | ATSFormer (501) | **86.95 ± 0.32** | **87.51 ± 2.63** | 86.31 ± 2.73 | **73.97 ± 0.67** | **94.81 ± 0.35** |
|  | ERNIE-RNA (501) | 84.43 ± 0.67 | 83.00 ± 3.60 | 85.93 ± 2.42 | 69.04 ± 1.13 | 91.85 ± 0.75 |
|  | Nucleotide Transformer (501) | 75.80 ± 0.58 | 71.88 ± 1.84 | 79.66 ± 2.13 | 51.75 ± 1.22 | 83.30 ± 0.65 |
|  | Grover (501) | 77.82 ± 0.70 | 71.79 ± 1.79 | 83.87 ± 1.09 | 56.08 ± 1.19 | 85.80 ± 0.43 |
|  | iMRM (41) | 86.45 ± 0.30 | 86.44 ± 0.83 | 86.49 ± 1.22 | 72.92 ± 0.60 | 94.45 ± 0.28 |
|  | RDDSVM (101) | 79.97 ± 1.39 | 71.52 ± 2.30 | **88.43 ± 1.25** | 60.84 ± 2.63 | 87.83 ± 1.29 |
|  | PreAIS (101) | 79.62 ± 1.13 | 82.16 ± 1.66 | 77.08 ± 0.70 | 59.32 ± 2.29 | 87.67 ± 0.74 |
|  | MRM-BERT (101) | 81.85 ± 9.80 | 82.00 ± 8.82 | 81.69 ± 11.22 | 63.75 ± 19.66 | 88.18 ± 10.69 |
|  | MultiRM (51) | 77.39 ± 1.03 | 74.33 ± 2.47 | 80.41 ± 1.69 | 54.89 ± 2.02 | 84.27 ± 1.01 |
| m5C | ATSFormer (501) | **89.09 ± 0.29** | **90.37 ± 2.76** | 87.80 ± 2.50 | **78.31 ± 0.63** | **95.94 ± 0.23** |
|  | ERNIE-RNA (501) | 86.74 ± 1.16 | 82.14 ± 4.15 | **91.34 ± 2.33** | 73.94 ± 1.96 | 94.37 ± 0.73 |
|  | Nucleotide Transformer (501) | 79.32 ± 0.70 | 77.05 ± 1.66 | 81.58 ± 0.94 | 58.71 ± 1.38 | 87.13 ± 0.55 |
|  | Grover (501) | 80.81 ± 0.47 | 77.03 ± 1.73 | 84.59 ± 1.00 | 61.82 ± 0.84 | 88.80 ± 0.51 |
|  | iMRM (41) | 88.40 ± 0.34 | 89.96 ± 0.91 | 86.84 ± 0.76 | 76.85 ± 0.70 | 95.64 ± 0.25 |
|  | RDDSVM (101) | 82.96 ± 0.36 | 82.28 ± 0.77 | 83.65 ± 0.63 | 65.94 ± 0.73 | 90.49 ± 0.16 |
|  | PreAIS (101) | 82.47 ± 0.29 | 83.39 ± 0.94 | 81.55 ± 0.67 | 64.95 ± 0.57 | 90.62 ± 0.27 |
|  | MRM-BERT (101) | 75.84 ± 13.43 | 84.16 ± 9.41 | 68.15 ± 33.52 | 53.66 ± 23.57 | 87.35 ± 5.09 |
|  | MultiRM (51) | 80.60 ± 0.54 | 81.42 ± 1.71 | 79.76 ± 2.34 | 61.20 ± 1.16 | 88.57 ± 0.46 |
| m7G | ATSFormer (501) | **85.72 ± 0.52** | 88.96 ± 2.50 | 82.47 ± 1.68 | **71.65 ± 1.18** | **93.59 ± 0.43** |
|  | ERNIE-RNA (501) | 80.91 ± 1.57 | 73.50 ± 4.50 | **88.33 ± 3.91** | 62.72 ± 3.15 | 89.30 ± 1.40 |
|  | Nucleotide Transformer (501) | 73.09 ± 0.88 | 71.09 ± 2.10 | 75.13 ± 1.02 | 46.26 ± 1.69 | 80.56 ± 1.01 |
|  | Grover (501) | 77.65 ± 0.83 | 75.31 ± 1.83 | 79.93 ± 3.01 | 55.38 ± 1.77 | 85.26 ± 0.86 |
|  | iMRM (41) | 83.42 ± 0.35 | 87.09 ± 0.75 | 79.75 ± 0.97 | 67.03 ± 0.63 | 91.92 ± 0.35 |
|  | RDDSVM (101) | 77.11 ± 1.22 | 74.09 ± 1.48 | 80.12 ± 1.17 | 54.30 ± 2.43 | 84.97 ± 0.62 |
|  | PreAIS (101) | 77.89 ± 0.50 | 78.07 ± 1.43 | 77.72 ± 1.40 | 55.80 ± 1.03 | 85.73 ± 0.59 |
|  | MRM-BERT (101) | 84.79 ± 0.73 | **90.78 ± 3.86** | 78.82 ± 4.75 | 70.35 ± 1.07 | 92.10 ± 0.68 |
|  | MultiRM (51) | 75.49 ± 1.25 | 75.19 ± 2.10 | 75.71 ± 1.12 | 50.95 ± 2.44 | 82.81 ± 0.88 |

**Supplementary Table 4. Performance comparison under independent data splits on four benchmark datasets.**

| Dataset | Methods | ACC(%) | Se(%) | Sp(%) | MCC(%) | AUC(%) |
| --- | --- | --- | --- | --- | --- | --- |
| A-to-I | ATSFormer (501) | **92.79** | 90.43 | **95.16** | **85.68** | **97.16** |
|  | ERNIE-RNA (501) | 89.04 | 88.21 | 89.87 | 78.09 | 93.48 |
|  | Nucleotide Transformer (501) | 73.81 | 69.97 | 77.65 | 47.76 | 80.00 |
|  | Grover (501) | 88.14 | 86.18 | 90.11 | 76.34 | 93.03 |
|  | iMRM (41) | 89.97 | 89.79 | 90.16 | 79.95 | 95.68 |
|  | RDDSVM (101) | 87.73 | 87.83 | 87.62 | 75.45 | 91.70 |
|  | PreAIS (101) | 87.54 | 88.45 | 86.63 | 75.09 | 92.82 |
|  | MRM-BERT (101) | 90.76 | **91.52** | 90.00 | 81.53 | 94.45 |
|  | MultiRM (51) | 81.48 | 85.90 | 77.09 | 63.17 | 87.62 |
| m1A | ATSFormer (501) | **85.62** | **84.56** | 86.68 | **71.25** | **94.18** |
|  | ERNIE-RNA (501) | 83.88 | 78.76 | 89.00 | 68.12 | 91.52 |
|  | Nucleotide Transformer (501) | 72.88 | 68.15 | 77.61 | 45.96 | 80.87 |
|  | Grover (501) | 75.10 | 69.88 | 80.31 | 50.47 | 83.40 |
|  | iMRM (41) | 84.65 | 84.17 | 85.14 | 69.31 | 92.50 |
|  | RDDSVM (101) | 80.50 | 70.46 | **90.54** | 62.27 | 88.54 |
|  | PreAIS (101) | 80.41 | 76.06 | 84.75 | 61.04 | 88.55 |
|  | MRM-BERT (101) | 66.31 | 55.41 | 77.22 | 33.43 | 71.56 |
|  | MultiRM (51) | 52.78 | 82.01 | 23.23 | 6.49 | 60.87 |
| m5C | ATSFormer (501) | **89.50** | 88.14 | **90.86** | **79.03** | **95.94** |
|  | ERNIE-RNA (501) | 86.56 | 84.62 | 88.49 | 73.17 | 94.27 |
|  | Nucleotide Transformer (501) | 77.64 | 77.77 | 77.50 | 55.27 | 85.50 |
|  | Grover (501) | 78.73 | 75.66 | 81.81 | 57.58 | 87.31 |
|  | iMRM (41) | 85.59 | **88.93** | 82.25 | 71.34 | 94.05 |
|  | RDDSVM (101) | 82.25 | 81.11 | 83.39 | 64.52 | 90.08 |
|  | PreAIS (101) | 82.21 | 79.53 | 84.89 | 64.50 | 90.60 |
|  | MRM-BERT (101) | 71.22 | 63.62 | 78.82 | 42.94 | 78.18 |
|  | MultiRM (51) | 56.88 | 69.37 | 44.30 | 14.09 | 59.04 |
| m7G | ATSFormer (501) | **86.06** | **90.44** | 81.68 | **72.41** | **93.45** |
|  | ERNIE-RNA (501) | 80.49 | 70.53 | **90.44** | 62.22 | 88.97 |
|  | Nucleotide Transformer (501) | 70.82 | 62.57 | 79.07 | 42.22 | 78.29 |
|  | Grover (501) | 75.65 | 76.00 | 75.31 | 51.31 | 83.70 |
|  | iMRM (41) | 85.15 | **90.44** | 79.86 | 70.70 | 92.70 |
|  | RDDSVM (101) | 76.79 | 76.11 | 77.47 | 53.59 | 84.89 |
|  | PreAIS (101) | 77.19 | 80.20 | 74.18 | 54.48 | 85.37 |
|  | MRM-BERT (101) | 66.04 | 61.09 | 70.99 | 32.24 | 72.32 |
|  | MultiRM (51) | 66.16 | 71.14 | 61.47 | 32.68 | 71.97 |

**2.2 Model Efficiency Study**

Detailed effects of input sequence length, the number of sampling tokens, and the number of Transformer sampling layers on model performance and computational efficiency are presented in Supplementary Tables 5–7, respectively.

**Supplementary Table 5. Comparison of model performance and token retention rates in the sampling layers under different sequence length settings.**

| Length | Performance Metrics(%) | | | | | Token Retention Rates (%) | | |
| --- | --- | --- | --- | --- | --- | --- | --- | --- |
|  | ACC | Se | Sp | MCC | AUC | Fourth layer | Fifth layer | Sixth layer |
| 51nt | 91.82 ± 0.20 | 89.52 ± 0.59 | 94.13 ± 0.44 | 83.74 ± 0.38 | 96.48 ± 0.12 | 73.73 | 55.77 | 43.73 |
| 101nt | 92.38 ± 0.32 | 90.52 ± 0.34 | 94.24 ± 0.70 | 84.82 ± 0.65 | 96.68 ± 0.17 | 74.21 | 53.87 | 40.50 |
| 301nt | 92.47 ± 0.22 | 90.15 ± 0.22 | **94.80 ± 0.34** | 85.04 ± 0.44 | 96.75 ± 0.19 | 67.40 | 45.51 | 31.54 |
| 501nt | **92.61 ± 0.20** | **90.90 ± 0.47** | 94.32 ± 0.71 | **85.28 ± 0.43** | 96.89 ± 0.14 | 62.18 | 40.00 | 26.45 |
| 701nt | 92.52 ± 0.24 | 90.25 ± 0.60 | 94.79 ± 0.44 | 85.13 ± 0.47 | 96.84 ± 0.14 | 61.50 | 39.02 | 25.52 |
| 901nt | 92.53 ± 0.24 | 90.27 ± 0.45 | 94.79 ± 0.39 | 85.15 ± 0.48 | 96.84 ± 0.13 | 57.22 | 36.57 | 23.65 |
| 1001nt | 92.53 ± 0.16 | 90.47 ± 0.40 | 94.59 ± 0.66 | 85.13 ± 0.36 | **96.91 ± 0.20** | 49.40 | 30.28 | 19.30 |

**Supplementary Table 6. Comparison of model performance and token retention rates in the sampling layers under different sampling token numbers.**

| Keep_k | Performance Metrics(%) | | | | | Token Retention Rates (%) | | |
| --- | --- | --- | --- | --- | --- | --- | --- | --- |
|  | ACC | Se | Sp | MCC | AUC | Fourth layer | Fifth layer | Sixth layer |
| 32 | 92.51 ± 0.20 | 90.81 ± 0.66 | 94.21 ± 0.73 | 85.08 ± 0.40 | 96.83 ± 0.20 | 18.76 | 13.30 | 9.52 |
| 64 | 92.50 ± 0.19 | 90.79 ± 0.90 | 94.21 ± 0.65 | 85.07 ± 0.36 | 96.79 ± 0.16 | 33.45 | 22.33 | 15.38 |
| 128 | 92.55±0.16 | 90.78±0.85 | **94.33±0.75** | 85.17 ± 0.30 | 96.86±0.13 | 55.01 | 35.60 | 23.72 |
| 256 | **92.61 ± 0.20** | **90.90 ± 0.47** | 94.32 ± 0.71 | **85.28 ± 0.43** | **96.89 ± 0.14** | 62.18 | 40.00 | 26.45 |
| 512 | **92.61 ± 0.20** | **90.90 ± 0.47** | 94.32 ± 0.71 | **85.28 ± 0.43** | **96.89 ± 0.14** | 62.18 | 40.00 | 26.45 |
| 1024 | **92.61 ± 0.20** | **90.90 ± 0.47** | 94.32 ± 0.71 | **85.28 ± 0.43** | **96.89 ± 0.14** | 62.18 | 40.00 | 26.45 |
| 2048 | **92.61 ± 0.20** | **90.90 ± 0.47** | 94.32 ± 0.71 | **85.28 ± 0.43** | **96.89 ± 0.14** | 62.18 | 40.00 | 26.45 |

**Supplementary Table 7. Comparison of model performance and token retention rates under different numbers of sampling layers.**

| Sampling layers | Performance Metrics(%) | | | | | Token Retention Rates (%) | | | | | |
| --- | --- | --- | --- | --- | --- | --- | --- | --- | --- | --- | --- |
|  | ACC | Se | Sp | MCC | AUC | Sixth | Fifth | Fourth | Third | Second | First |
| 1 | 92.59 ± 0.27 | 90.89 ± 0.30 | 94.28 ± 0.79 | 85.23 ± 0.57 | 96.81 ± 0.21 | 65.10 | _ | **_** | **_** | **_** | **_** |
| 2 | 92.46 ± 0.27 | 90.44 ± 0.65 | 94.50 ± 0.65 | 85.01 ± 0.54 | 96.85 ± 0.19 | 65.49 | 42.49 | _ | _ | _ | _ |
| 3 | **92.61 ± 0.20** | **90.90 ± 0.47** | 94.32 ± 0.71 | **85.28 ± 0.43** | **96.89 ± 0.14** | 62.18 | 40.00 | 26.45 | _ | _ | _ |
| 4 | 92.53 ± 0.17 | 90.86 ± 0.23 | 94.19 ± 0.25 | 85.10 ± 0.35 | 96.86 ± 0.16 | 54.10 | 33.63 | 21.64 | 14.87 | _ | _ |
| 5 | 92.49 ± 0.17 | 90.36 ± 0.54 | **94.61 ± 0.70** | 85.05 ± 0.36 | 96.75 ± 0.13 | 45.25 | 26.06 | 16.41 | 11.34 | 8.18 | _ |
| 6 | 92.41 ± 0.17 | 90.71 ± 0.16 | 94.10 ± 0.38 | 84.87 ± 0.35 | 96.70 ± 0.07 | 43.67 | 21.13 | 13.51 | 9.39 | 6.92 | 5.32 |

**2.3 Ablation Study**

Detailed comparisons of ablation study results and the performance of different sampling methods are presented in Supplementary Tables 8 and 9, respectively.

**Supplementary Table 8: Performance comparison of ATSFormer and ablation models.**

| Methods | ACC(%) | Se(%) | Sp(%) | MCC(%) | AUC(%) |
| --- | --- | --- | --- | --- | --- |
| ATSFormer | **92.61 ± 0.20** | **90.90 ± 0.47** | 94.32 ± 0.71 | **85.28 ± 0.43** | **96.89 ± 0.14** |
| w/o ATS | 92.56 ± 0.27 | 90.73 ± 0.57 | 94.39 ± 0.83 | 85.18 ± 0.56 | 96.85 ± 0.18 |
| w/o CNN | 91.73 ± 0.28 | 90.45 ± 0.69 | 93.02 ± 0.39 | 83.50 ± 0.56 | 96.60 ± 0.26 |
| w/o CNNoneLayer | 92.42 ± 0.15 | 90.24 ± 0.52 | **94.60 ± 0.40** | 84.92 ± 0.29 | 96.83 ± 0.11 |
| w/o Transformer | 87.79 ± 0.12 | 87.09 ± 0.74 | 88.49 ± 0.87 | 75.59 ± 0.25 | 92.58 ± 0.16 |
| w/o Positional Encoding | 87.67 ± 0.16 | 86.70 ± 0.67 | 88.64 ± 0.55 | 75.36 ± 0.31 | 92.40 ± 0.27 |

**Supplementary Table 9: Performance comparison of different sampling methods.**

| Methods | ACC(%) | Se(%) | Sp(%) | MCC(%) | AUC(%) |
| --- | --- | --- | --- | --- | --- |
| Multinomial | **92.61 ± 0.20** | **90.90 ± 0.47** | 94.32 ± 0.71 | **85.28 ± 0.43** | **96.89 ± 0.14** |
| Inverse Transform | 92.56 ± 0.17 | 90.32 ± 0.47 | **94.79 ± 0.43** | 85.20 ± 0.35 | 96.85 ± 0.11 |
| TopK | 92.38 ± 0.15 | 90.17 ± 0.42 | 94.59 ± 0.44 | 84.85 ± 0.29 | 96.65 ± 0.10 |

**2.4 Motifs Identified by ATSFormer Using In Silico Saturation Mutagenesis**

In RNA modification recognition tasks, a motif typically refers to a sequence fragment with specific characteristics and often serves as an indicator of protein-binding sites or functional regions. We employed the in silico saturation mutagenesis (ISM) method to systematically analyze the importance matrices generated by the ATSFormer model, thereby identifying key motifs associated with A-to-I editing (Supplementary Figure 1). This analysis was performed on the four randomly selected A-to-I sequences investigated in Section 4.5 of the main text.

**Supplementary Figure 1. Motifs identified by ATSFormer using in silico saturation mutagenesis.**
